# Overwintering potential of the Mediterranean fruit fly (Diptera: Tephritidae) in Austria

**DOI:** 10.1093/jee/toae180

**Published:** 2024-08-09

**Authors:** Matthias Wernicke, Alois Egartner, Sylvia Blümel, Cleopatra A Moraiti, Nikos T Papadopoulos

**Affiliations:** Austrian Agency for Health and Food Safety (AGES), Institute for Sustainable Plant Production (NPP), Vienna, Austria; Austrian Agency for Health and Food Safety (AGES), Institute for Sustainable Plant Production (NPP), Vienna, Austria; Austrian Agency for Health and Food Safety (AGES), Institute for Sustainable Plant Production (NPP), Vienna, Austria; Department of Agriculture, Crop Production and Rural Environment, Laboratory of Entomology and Agricultural Zoology, University of Thessaly, Volos, Greece; Department of Agriculture, Crop Production and Rural Environment, Laboratory of Entomology and Agricultural Zoology, University of Thessaly, Volos, Greece

**Keywords:** Tephritids, cold tolerance, establishment, range expansion, insect ecology

## Abstract

The Mediterranean fruit fly (medfly), *Ceratitis capitata* (Wiedemann), one of the most important invasive pests of fresh fruits and vegetables from the coastal Mediterranean habitats, is expanding its current geographic distribution to cooler more temperate areas of Europe. Every year since 2010 the fly is detected in the area of Vienna, Austria. However, whether it can establish permanent populations is not known. In this current paper, the capacity of *C. capitata* to overwinter in Vienna, Austria (48.1° northern latitude) was studied over 2 consecutive winter seasons (2020–2022). Overwintering trials with different life stages (larva, pupa, and adult) of *C. capitata* were performed in the open field and in the protected environment of a basement without a heating system. Control flies were kept under constant conditions in a climate chamber (25 °C, 60% RH, 14:10 L:D). Our data showed that no life stage of the Mediterranean fruit fly was able to survive the Austrian winter in the open field. However, in the protected environment *C. capitata* outlived the winter months in all studied life stages at least in small numbers and several surviving females were able to lay eggs at the time of the following fruiting season. Implications of these findings for the ongoing geographic range expansion of the pest in temperate European countries are discussed.

## Introduction

Over the last decades, the introduction and establishment of tropical pests in countries of the temperate zone are rising. Global warming is one of the key factors responsible for this development. Warmer temperatures during the winter period lead to an expansion of the habitable zone northwards for pests from tropical areas, making a permanent establishment more likely (Szyniszewska and Tatem 2014, Gilioli et al. 2022, Szyniszewska et al. 2024). Another contributing factor is the global expansion of human travel and trade activities, which increase the chances of an accidental introduction of a non-native pest. A prominent example for that is the Mediterranean fruit fly (medfly), *Ceratitis capitata* (Wiedemann), which is considered one of the most important pests for fresh fruit production worldwide. Native to sub-Saharan Africa, medfly has spread successfully in many tropical and temperate areas of the world (White and Elson-Harris 1992, Bonizzoni et al. 2004, Deschepper et al. 2021, EPPO 2024). It is highly polyphagous with over 350 host plants from more than 70 families (Papadopoulos et al. 2001, Badii et al. 2015, Liquido et al. 2020, EPPO 2024), including crop species with high importance for the European and Mediterranean Plant Protection Organization (EPPO) region like *Citrus* spp. L., *Prunus* spp. L. (peach (*P. persica* (L.) Batsch), apricot (*P. armeniaca* L.)), pear (*Pyrus communis* L.) (De Meyer et al. 2002) and apple (*Malus* spp. MILL.) (Duyck et al. 2008), all of which, with the exception of citrus, are cultivated in Austria.

Due to adaptive and plastic responses to a range of thermal and hydric conditions, as well as the extreme polyphagy, medfly is capable of inhabiting a wide range of environments ranging from tropic, and deserting to temperate (Duyck and Quilici 2002, Grout and Stoltz 2007, Nyamukondiwa and Terblanche 2009, Nyamukondiwa et al. 2010, Weldon et al. 2016). Medfly is categorized as a quarantine pest in various countries, such as the United States of America (APHIS 2024), Mexico (EPPO 2024), Japan (MAFF 2022), and China (IPPC 2021). An introduction (detection and/or establishment) into a novel area has severe economic consequences on the fruit production, ranging from the implementation of trade barriers and expensive phytosanitary programs to severe direct crop losses (Papadopoulos 2014, Badii et al. 2015). Previous introductions into pest-free areas, like North America, required extensive monitoring and eradication programs and even preventive releases of sterile males for several decades (Siebert and Cooper 1995, Papadopoulos et al. 2013, Szyniszewska and Tatem 2014, State of California—CDFA 2023). Propagule pressure resulting from intense fruit trade (e.g., seasonal importation of citrus in central Europe from Mediterranean countries) and travelers’ movement from endemic to medfly-free areas facilitate interception, incursion, and establishment events (Meixner et al. 2002, Israely et al. 2004, Peñarrubia-María et al. 2012).

The geographic distribution of a newly introduced species depends on abiotic (temperature, relative humidity, and precipitation) and biotic (host plants and natural enemies) factors. Thermal thresholds and the availability of host plants are supposed to be crucial factors for the establishment of medfly in colder regions (Papadopoulos et al. 1996, 1998, 2001, Israely et al. 2004, Peñarrubia-María et al. 2012). Areas south of the 41° northern latitude were suggested as the northern European limits of *C. capitata* distribution (Fischer-Colbrie and Bush-Petersen 1989, Papadopoulos et al. 1996, 1998). However, in recent years, the records of medfly in regions above this limit, including northern Italy (Rigamonti et al. 2004), France, and Germany (EPPO 2024) increased, raising the question of the origin of those detections (Gilioli et al. 2022, König et al. 2022).

Over the last century, several detections of *C. capitata* in Austria (recurrent catches and reports of fruit infestations) were reported (Watzl 1931, 1932, Böhm 1958, Glaeser 1979). Furthermore, monitoring activities since 2010 resulted in annual catches of the pest in Vienna, Austria (Egartner et al. 2019). While the origin of these flies remains unknown, the repeated detections of *C. capitata* individuals in Austria may be linked to recurrent entries of juveniles with infested fruits. However, the detections in consecutive years at the same sites support the theory that in certain Viennese areas the catches might have been the result of specimens surviving the winter period.

There are 2 main hypotheses for the recurrent occurrences of medfly in regions above the northern limit. First, *C. capitata* is repeatedly introduced to these areas through natural distribution from nearby favorable areas (Peñarrubia-María et al. 2012) or international trade and traveling (Siebert and Cooper 1995, EUROPHYT 2015, 2017). The second hypothesis is that, although medfly is a nondiapausing tephritid, it has managed to adapt to cold climate conditions and is able to survive the winter period in those regions (i.e., as larva within apples; Papadopoulos et al. 1996, 1998). An alternative hypothesis is that, in regions with freezing temperatures, medfly is not overwintering in the open field, but in more favorable conditions that provide protection from outdoor climate (Rigamonti 2004).

The aim of the present study was to investigate the overwintering potential of various life stages of *C. capitata* in Austria. Experiments were designed to answer the research questions if (i) medfly is able to survive the conditions of an Austrian winter, and if so (ii) at which life stage (larva, pupa, and adult) and environmental conditions.

## Materials and Methods

### Test Sites, Insects, and Design

The ability of medfly to overwinter in Vienna, Austria was examined considering 2 overwintering scenarios: (i) open field (open field conditions) and (ii) a man-made shelter (urban conditions; non-heated basement in urban area). Both overwintering sites were located on the premises of the AGES facility, which is situated in a residential area at the periphery of Vienna, Austria (48°15ʹ13″N, 16°28ʹ56″E, 160 m above sea level). The compound comprises agricultural land and orchards (apple, apricot, and plum) for field trials, and is surrounded by urban settlements. This location was chosen as the trial site for 2 reasons: first, because 99% of all medfly catches in the national survey program were made in Vienna (Egartner et al. 2019). And second, since abiotic factors (temperature, relative humidity, and precipitation) are presumably the most limiting factor for a successful overwintering of *C. captitata* at this latitude and taking into consideration the prevailing 4 climate zones in Austria, we assumed that the urban area of Vienna would provide the most favorable climate conditions for *C. capitata* survival. The 4 main climate zones in Austria comprise the alpine zone in the West and South, the alpine pre-land in the North, the Illyrian zone in the South–East and the Pannonian zone, including Vienna, in the East of Austria. The first 3 climate zones are shaped by the influence of mountains with long, cold winters, and high precipitation. In contrast, the fourth climate zone (Pannonian zone) provides milder winters with less precipitation than the rest of the country. Additionally, the urbanization of Vienna leads to higher average temperatures compared with the nonurban areas of the Pannonian zone (Diamond and Martin 2020). Based on those factors, it was concluded that if the establishment of a *C. capitata* population in Austria is possible at all, the urban area of Vienna would provide the best conditions for it.

For the overwintering scenario under open field conditions, medflies were exposed to the naturally occurring Austrian winter climate in a roofed building (to protect flies from precipitation and wind) with double-netted walls (mesh size: 1.5 mm) surrounded by agricultural land (48°15ʹ24″N, 16°28ʹ59″E; 160 m above sea level). Cages for the overwintering trials of adults and larvae were placed inside the building, while cages for overwintering of pupae were buried next to it into the soil.

For the scenario in urban conditions, it was hypothesized that medfly may survive the winter period in a protected habitat such as a basement. This can be achieved either by actively seeking refuge as an adult in autumn as other insects do (Cullum et al. 2020) or by being introduced to these environments as an egg or larva in infested fruit. In Austria, it is common to store apples and pears in unheated cellars over the winter. Additionally, small allotments in the urban area, where people grow fruits, are widespread in Vienna. To test this hypothesis, trials in urban conditions were conducted in a storage room without windows and an active heating system, which was located in the basement of the main building of the AGES facility. As basements can provide a wide spectrum of climate conditions, we chose to use a common set of conditions in Austria: an unheated basement where the climate inside is thus partly affected by the climate conditions outside.

All trials for the control group (larva, pupa, and adult) were conducted in a walk-in climate chamber (25 °C, 60% RH, 14:10 L:D), which is from now on only referred to as the “control.” According to the Köppen–Geiger climate classification Vienna is categorized as Dfb, a cold temperate fully humid climate with warm summers (Beck et al. 2018). Temperature and humidity during the trial periods were recorded hourly with climate data loggers (Tinytag, Gemini Data Loggers, Chichester, United Kingdom) at the test sites next to the tested specimens.

All flies used in this study were obtained from a laboratory colony (25 °C, 60% RH, 14:10 L:D) originated from wild flies that were obtained from infested fruits (apple, apricot, peach), collected in Viennese allotments in the year prior to the respective trial season (June–August). It is unclear, if these fruits were infested by medflies that were accidently introduced by import/travel activities to the area or by an established Viennese population.

Emerging adults from infested fruits were kept under controlled climate conditions (25 °C, 60% RH, 14:10 L:D) in insect cages (Bugdorm-1, 30 × 30 × 30 cm, polypropylene, MegaView Science Co. Ltd, Taiwan), supplied with water and adult food (hydrolyzed yeast, sugar, water; ratio 1:4:5; ad libitum) until the start of the trials. Eggs of new generations were collected using artificial oviposition devices (Ø 6 cm domes, red, pre-punctured plastic hollow hemispheres). Fresh orange juice soaked on cotton pads positioned beneath the domes was used as an oviposition stimulant. Collected eggs were transferred to larvae rearing medium soaked on cotton pads (based on Boller 1958). Resulting pupae were transferred to rearing cages. F_6_ and F_7_ laboratory-reared flies were used in all overwintering trials.

The methodology for the adult, larvae, and pupae trials was adapted from Papadopoulos et al. (1996, 1998). In brief, the overwintering potential of those life stages were tested by exposing adults in cages, pupae covered in soil, and larvae as infested apples to the climatic conditions at the trial site. The methodology to evaluate the lifespan and reproduction potential of adults obtained from the larvae and pupae trials was based on the work of Diamantidis et al. (2009) whereby a female and a male medfly were paired immediately after emergence and placed into a cage containing adult diet, water, and oviposition substrate (pre-punctured plastic dome). Trials were carried out in 2 consecutive winter seasons (2020/2021, 2021/2022) and started in the middle of October. At each site, trials were conducted at 3 different dates in fortnight intervals (i.e., mid of October, end of October, and mid of November). All life stages (larva, pupa, and adult) were tested at both test sites and at each establishment date. Trials lasted until the death of the test specimens, or at least until the end of June of the following year.

### Adult Overwintering Trials

Groups of 10, 7–10 d old adults (5♂♂ + 5♀♀) kept in plastic cages (C-Box XS+, 19 × 33.5 × 12 cm, polypropylene, transparent, modification: ventilated lid and sides, KIS, Ormelle, Italy) were exposed at the test sites. Ten cages were used per test site on each date (3 dates) (i.e., 30 cages in each of the 2 test sites and 60 cages with 600 flies in total). Five cages per date were used for the control. Fresh water and adult food were provided ad libitum and refreshed weekly. Mortality was determined by visual inspection twice a week until the death of the last adult specimen, involving a gentle physical stimulus and observation of adult response like tightened legs/abdomen and the change of eye color from green/blue to brown/black. Dead flies were removed from cages, sexed, and monitored for an additional 24 h under laboratory conditions to confirm death.

### Pupae Overwintering Trials

Two treatments, adapted to the test sites, were used to evaluate the overwintering capacity of *C. capitata* pupae. In urban conditions, it was assumed that since basements normally have no soil, pupae will either be found on top of a surface or at most be covered by a thin layer of substrate. We examined this version by using plastic cups (polypropylene, 11 cm height, Ø 10cm, sealed with insect net (gaze) fixed by rubber band) and putting 20 pupae (2–5 d after pupation) on top of 4 cm dry soil in the bottom of each cup. Afterwards, pupae were covered with an additional layer of 1 cm thick sand.

In the open field, it was assumed that pupae, that are buried in the soil, had a higher survival chance than those close to the surface, where they were constantly exposed to lower air temperature than that of the soil. We used emptied water bottles (polypropylene, 30 cm height, Ø 7.5 cm, 1 L Vol., Vöslauer Mineralwasser GmbH, Bad Vöslau, Austria) that were buried outside of the described shed 15 cm into the ground and protected from wild animals with a separate roof (0.5 m height) and lattice. In each bottle, we placed 20 pupae on 5 cm of soil and then buried the pupae with an additional 8 cm of soil and 2 cm of sand on top (i.e., 10 cm in total). Ten replicates (cups/bottles) per date (3 dates) and trial site (2 sites: open field/urban conditions) were established (i.e., 30 replicates per test site and 60 with 1,200 pupae in total), while 5 replicates were kept under constant laboratory conditions to serve as the control. Cups/bottles were inspected 2–3 times per week for adult emergence until the end of June of the consecutive year.

### Larvae Overwintering Trials

For the overwintering trials with *C. capitata* larvae, infested apples (*Malus domestica*, cultivar “Golden Delicious”; organic production from a medfly-free area) were placed at the test sites and monitored for larvae/pupae development. The infestation of the apples was achieved by placing 4 adults (2♂♂ + 2♀♀; older than 21 d to assure reproductive maturity) into a plastic cage (C-Box XS+, 19 × 33.5 × 12 cm, polypropylene, transparent, modification: ventilated lid, KIS, Ormelle, Italy) with 1 ripe apple (prior acclimatized to room temperature) for 24 h. Water and adult food were provided ad libitum. Infested apples were transferred into boxes (C-Box M+, 34 × 40 × 17 cm, transparent, polypropylene, modification: ventilated lid, KIS, Ormelle, Italy). Each box contained 10 infested apples placed on a grid above a thin layer of sand. After the oviposition process, apples were kept 48 h under rearing conditions to promote larval hatching before they were exposed at the test sites. For each establishment date (3 dates) and test site (2 sites), 7 boxes (i.e., 70 apples per date and site, 420 apples in total) were used. The control group, which was kept under controlled laboratory conditions, consisted of 3 boxes (total: 30 apples) for each establishment date. The sand of the boxes was sieved weekly for larvae/pupae until the end of June of the consecutive year. Collected pupae were placed into Petri dishes (Ø 90 mm, 25 mm height, polystyrene, modification: perforated lid, Semadeni, Ostermundigen, Switzerland) filled with 1 cm of sand and kept at the test sites.

To evaluate the survival of the larvae under open field conditions during the test period, a “Field control” was implemented for this test site in 2021/22. It consisted of 3 additional boxes (details as above but with 5 infested apples each instead of 10) per establishment date (3 dates), of which 1 box was removed every 6 weeks and kept under the climate conditions of the control group to monitor the progress of pupation and/or third instars leaving fruit to pupate to determine mortality patterns of immatures in the wild. Field control ended 18 weeks after the respective establishment date.

### Lifespan and Reproduction of Adults Obtained from the Larvae and Pupae Trials

Adults that were obtained from immatures (larvae and/or pupae) exposed to the overwintering sites were sexed and transferred in pairs of 1 male and 1 female into individual cages to record longevity and their ability to produce eggs. These cages were kept at their original overwintering site during this process. Only adults that had a maximum age of no longer than 3 d and that were from the same trial (pupae or larvae), test site, and establishment date were paired. Each cage (wide mouth bottle, Ø 10 cm, 9 cm height, 500 ml, transparent, polyethylene, modification: ventilated side; Semadeni, Ostermundigen, Switzerland) contained adult food, water, and an oviposition dome with oviposition stimulant (see above for details). To inhibit mold development on the cotton pads below the dome, methylparaben (0.3% solution, w/w, VWR Chemicals, Vienna, Austria) was added to the freshly pressed orange juice. Food, water, and oviposition stimulant were changed regularly. Cages and oviposition domes were inspected up to 3 times per week to record and remove laid eggs and register dead individuals. All cages were monitored until the death of all flies; the oviposition domes were inspected for eggs until the death of the female. The number of replicates (=cages with pair of flies) was dependent on the number of emerging adults from the overwintering trials of larvae or pupae, but was limited to a maximum number of 30 (first season) and 60 cages (second season) per test site, trial developmental stage (pupa/larva), and establishment date.

In total, due to the different survival rates, 90 (first season) and 180 cages (second season) were realized with adults from the overwintering of pupae trials at the test site urban, but 0 (first season) and 4 cages (second season) at open field. As far as overwintering of larvae trials were regarded, no cages could be established at the test site open field in either season, while at the test site urban the amount was 0 (first season) and 14 (second season).

### Statistical Analysis

The Cox proportional hazard model was used to assess (i) the effects of overwintering site, establishment date, sex, and their interactions on mortality patterns of overwintering adults, (ii) the effects of establishment date and sex on adult lifespan for adults emerged from overwintering pupae and pupae-to-adult developmental time for adults emerged from overwintering pupae, and (iii) the effect of establishment date on larval developmental time for overwintering larvae, (iv) the effect of establishment date on the duration of reproductive periods (pre-oviposition (days from adult hatch to first egg laid), oviposition (days from first to last egg laid) and post-oviposition (days from last egg laid to death)) of females emerged from overwintering pupae and (v) the effect of sex on lifespan of adults obtained from overwintering larvae. Signiﬁcant factors were entered into a multifactorial Cox regression model using a forward stepwise procedure for model selection. Non-signiﬁcant factors (Wald’s *t*-test) were excluded from the ﬁnal model. Binary logistic regression analysis was used to assess the effect of establishment date on survival patterns of overwintering larvae and pupae. The non-parametric Kruskal–Wallis test was used to assess the effect of establishment date on fecundity of females emerged from overwintering pupae. Statistical analysis was performed with SPSS*®* Statistics—Version 26 (IBM Corp. 2019).

## Results

### Climatic Conditions at the Test Sites

**Fig. 1.**
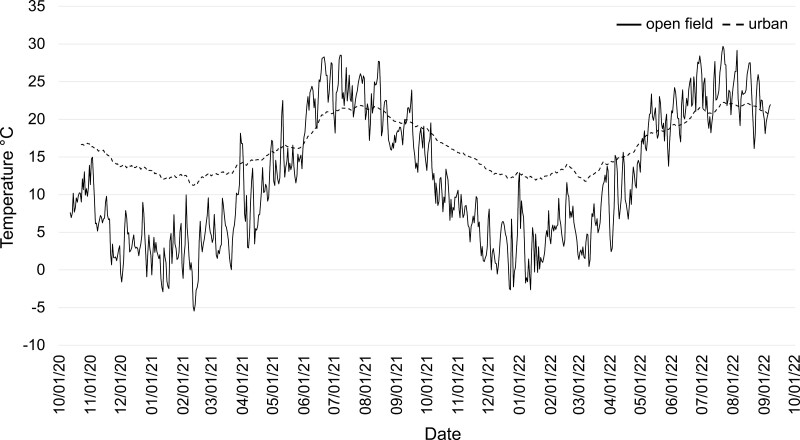
Mean temperature (°C) at the overwintering sites open field and urban in the seasons 2020/2021 and 2021/2022 in Vienna, Austria.

In open field conditions, the mean daily air temperature ranged from −5.5 to 28.3 °C in 2020/21 and −2.7 to 28.4 °C in 2021/22 in the trial period from October to June. During both seasons, air temperatures dropped below 0 °C on several days between the end of November and April of the following year (Fig. 1). In total, 54 frost days (=minimum daily temperature <0 °C) were counted during the first season (2020/21) and 56 during the second (2021/22). The mean daily relative humidity ranged from 20.8 to 100% and 28.5 to 100% in 2021/22 and 2021/22, respectively. The mean soil temperature in 10 cm depth (where pupae for overwintering trials were buried) ranged from −0.6 to 23.5 °C in 2020/21 and 1.3 to 23.2 °C in 2021/22. Seven frost days (soil) were counted in 2020/21 and none in 2021/22.

At the test site urban conditions, air temperatures fluctuated only slightly during the day (±0.5 °C). Mean daily temperature ranged from 11.2 to 21.9 °C in 2020/21 and 11.8 to 22.2 °C in 2021/22. The mean relative humidity varied between 34.6 and 80.6% in 2020/21, and 35.0 to 76.5% in 2021/22.

### Adults Overwintering Trials

**Fig. 2.**
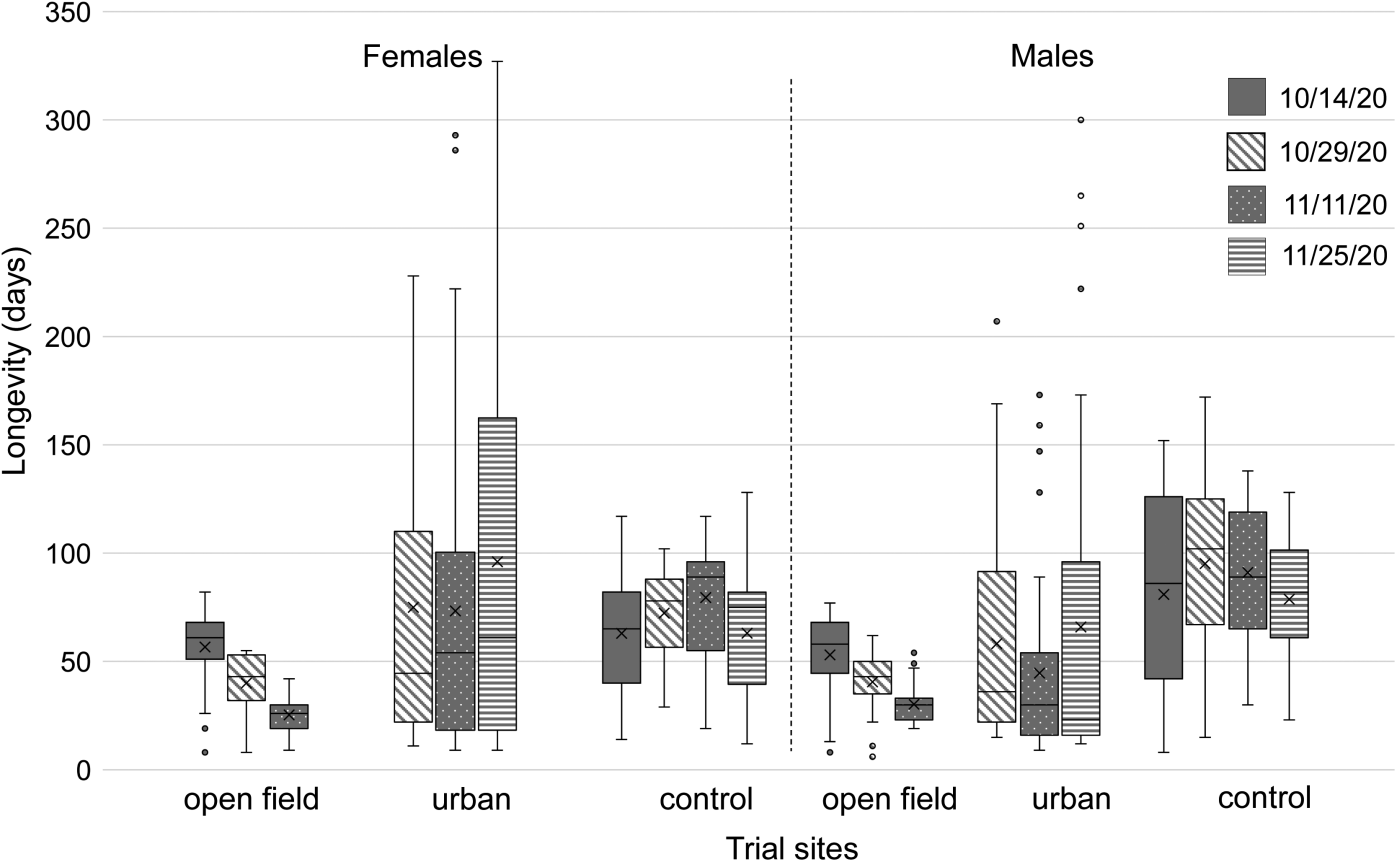
Longevity (days) of *Ceratitis capitata* females and males recorded in each overwintering site and for each establishment date for the trial season 2020/2021 in Vienna, Austria. Boxplots include the median (line), mean (x), and the first and third quartile; whiskers indicate the minimum/maximum value. A data point is considered an outlier (gray dots), if it exceeds a distance of ± the 1.5-fold interquartile range from the first/third quartile. Cox regression analysis considering overwintering site (open field, urban, and control), sex (males and females) and establishment date as predictors revealed that in 2020/2021 overwintering site (Wald *t*-test = 123.514, df = 2, *P* < 0.001), establishment date (*t* = 32.142, df = 3, *P* < 0.001) and establishment date by overwintering site (*t* = 189.242, df = 5, *P* < 0.001) as significant predictors for adult lifespan.

**Fig. 3.**
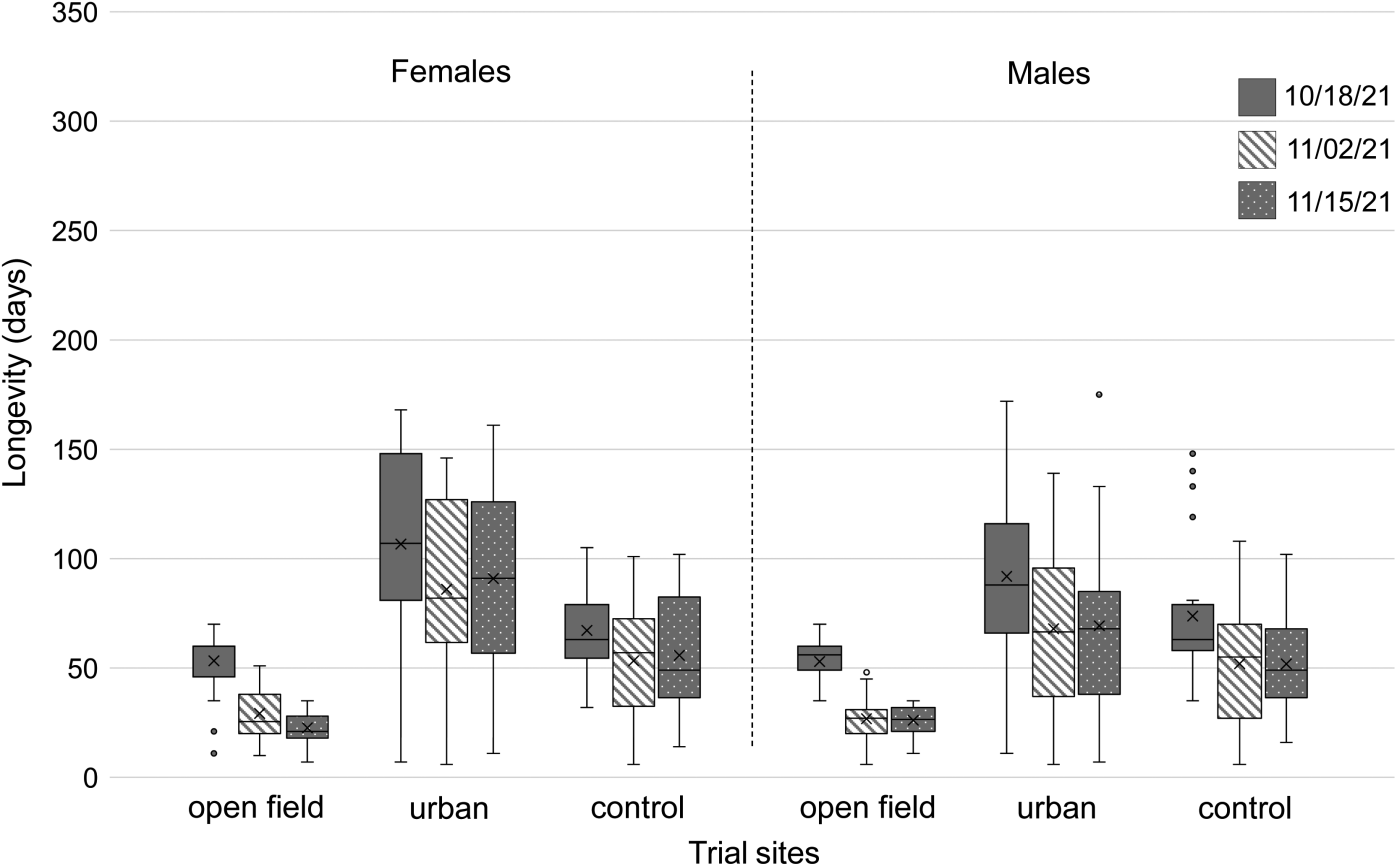
Longevity (days) of *Ceratitis capitata* females and males recorded in each overwintering site and for each establishment date for the trial season 2021/2022 in Vienna, Austria. Boxplots include the median (line), mean (x), and the first and third quartile; whiskers indicate the minimum/maximum value. A data point is considered an outlier (gray dots), if it exceeds a distance of ± the 1.5-fold interquartile range from the first/third quartile. Cox regression analysis considering overwintering site (open field, urban, and control), sex (males and females) and establishment date as predictors revealed that in 2021/2022 overwintering site (*t*-test = 305.733, df = 2, *P* < 0.001), establishment date (*t* = 12.646, df = 2, *P* = 0.002), sex (*t* = 6.078, df = 1, *P *= 0.014) and establishment date by overwintering site (*t* = 52.990, df = 4, *P* < 0.001) as significant predictors for adult lifespan.

On average, adult longevity was shorter in open field than under urban conditions (Table 1; Fig. 2, Fig. 3). Cox Regression analysis revealed the overwintering site as a significant predictor of adult longevity (*χ*^2^ = 19.472, df = 1, *P *< 0.001, and *χ*^2^ = 203.905, df = 1, *P *< 0.001 for the 2020/2021 and 2021/2022 season, respectively). Furthermore, the interaction between sex and the overwintering site was also a significant predictor of the adult survival during both seasons (*χ*^2^ = 9.978, df = 1, *P *= 0.002; *χ*^2^ = 8.624, df = 1, *P *= 0.003 for the 2020/2021 and 2021/2022 season, respectively).

**Table 1.** Effect of establishment date, overwintering site (open field, urban, and control), and sex on the average lifespan of *C. capitata* adults subject to overwintering trials in Vienna, Austria during the seasons 2020/2021 and 2021/2022

Average lifespan *Ceratitis capitata* adults (days ± SE)						
	Overwintering sites					
	Open field		Urban		Control	
Establishment dates	Males	Females	Males	Females	Males	Females
**2020** ^a^						
10/14/2020	53.0 ± 2.6	56.6 ± 2.2	-	-	81.0 ± 9.2	62.9 ± 5.6
10/29/2020	40.5 ± 2.0	40.0 ± 1.7	58.2 ± 6.8	74.9 ± 8.5	95.2 ± 8.2	72.3 ± 4.2
11/22/2020	30.2 ± 1.2	25.3 ± 1.0	44.6 ± 6.1	73.3 ± 9.9	91.0 ± 6.4	79.5 ± 5.6
11/25/2020	-	-	65.9 ± 10.7	96.0 ± 13.7	78.7 ± 6.4	63.1 ± 5.9
**2021** ^a^						
10/18/2021	53.0 ± 1.2	53.3 ± 1.7	91.9 ± 5.0	106.6 ± 6.1	73.8 ± 6.9	67.2 ± 3.6
11/02/2021	26.8 ± 1.3	29.2 ± 1.5	67.9 ± 5.2	86.0 ± 6.1	52.0 ± 5.3	53.3 ± 4.9
11/15/2021	26.1 ± 0.9	22.7 ± 0.9	69.3 ± 5.6	90.9 ± 6.2	51.9 ± 4.5	55.8 ± 5.7

^a^For statistical analysis see legends of Figs. 2 and 3 for the overwintering seasons 2020/2021 and 2021/2022, respectively.

In both seasons, mortality rates of adults increased in open field from the end of November onwards, the time that temperatures started to drop below 0 °C. All flies were dead in this site at the beginning of January (first season) or at the end of December (second season). Adult survival under open field conditions was also influenced by the establishment date of the trial (*χ*^2^ = 30.271, df = 3, *P *< 0.001; *χ*^2^ = 12.989, df = 2, *P *= 0.002, for the 2020/2021 and 2021/2022 season, respectively), with adults from earlier establishment dates expressing longer longevity than from the last one. Longevity of females and males under open field conditions was comparable in 2020/2021 and 2021/2022.

In contrast, under urban conditions, the average lifespan of females was in both seasons longer than that of males. At this site, adults from the last establishment date lived on average longer than from earlier ones in 2020/2021, but not in 2021/2022. In total, 21.3% (2020/2021) and 24.3% (2021/2022) of all adults under urban conditions managed to survive the Austrian winter and lived at least until the beginning of March of the following year. Additionally, 9.7% (2020/2021) and 0.7% (2021/2022) of all adults at this test site lived at least until the beginning of May (start of subsequent fruiting season). The death after the longest lifespan (oldest fly) was reported under urban conditions at day 327 of age (♀) in 2020/2021 and 175 d of age (♂) in 2021/2022 compared to the control with 172 d (♀) and 148 d (♀) in 2020/2021 and 2021/2022, respectively. Mean longevity of males was longer than that of females in control flies in both seasons.

### Pupae Overwintering Trials

The survival rate (adults emerged from exposed pupae) under open field conditions was very low, with only 0.3% in the first trial season and none in the second trial season. Adults that emerged in 2020/2021 were from the first establishment date (15.10.2020), but all died within a few weeks (2 December 2020 at the latest). In contrast, the survival rate of pupae under urban conditions in the first and second trial season was 95.7% and 93.2%, respectively. Comparable survival rates of 90.3% and 93.2% were recorded for the control group during the same period. The establishment date had no effect on the pupa survival under urban conditions in 2020/2021 (*χ*^2^ = 4.862, df = 2, *P *= 0.088), but the difference between the last and the earlier dates was significant in 2021/2022 (*χ*^2^ = 8.040, df = 2, *P *= 0.018).

Under urban conditions, pupae developmental duration recorded at adult emergence was on average longer at the later establishment dates than at the first one. In general, adults emerged after a few weeks under urban conditions compared to a few days at the control. While the establishment date was a significant predictor for pupae developmental duration (*χ*^2^ = 161.340, df = 2, *P *< 0.001; *χ*^2^ = 79.645, df = 2, *P *< 0.001, for the 2020/2021 and 2021/2022 season, respectively), sex was not (*χ*^2^ = 2.310, df = 1, *P *= 0.129; *χ*^2^ = 2.3394, df = 1, *P *= 0.122, for the 2020/2021 and 2021/2022 season, respectively).

**Fig. 4.**
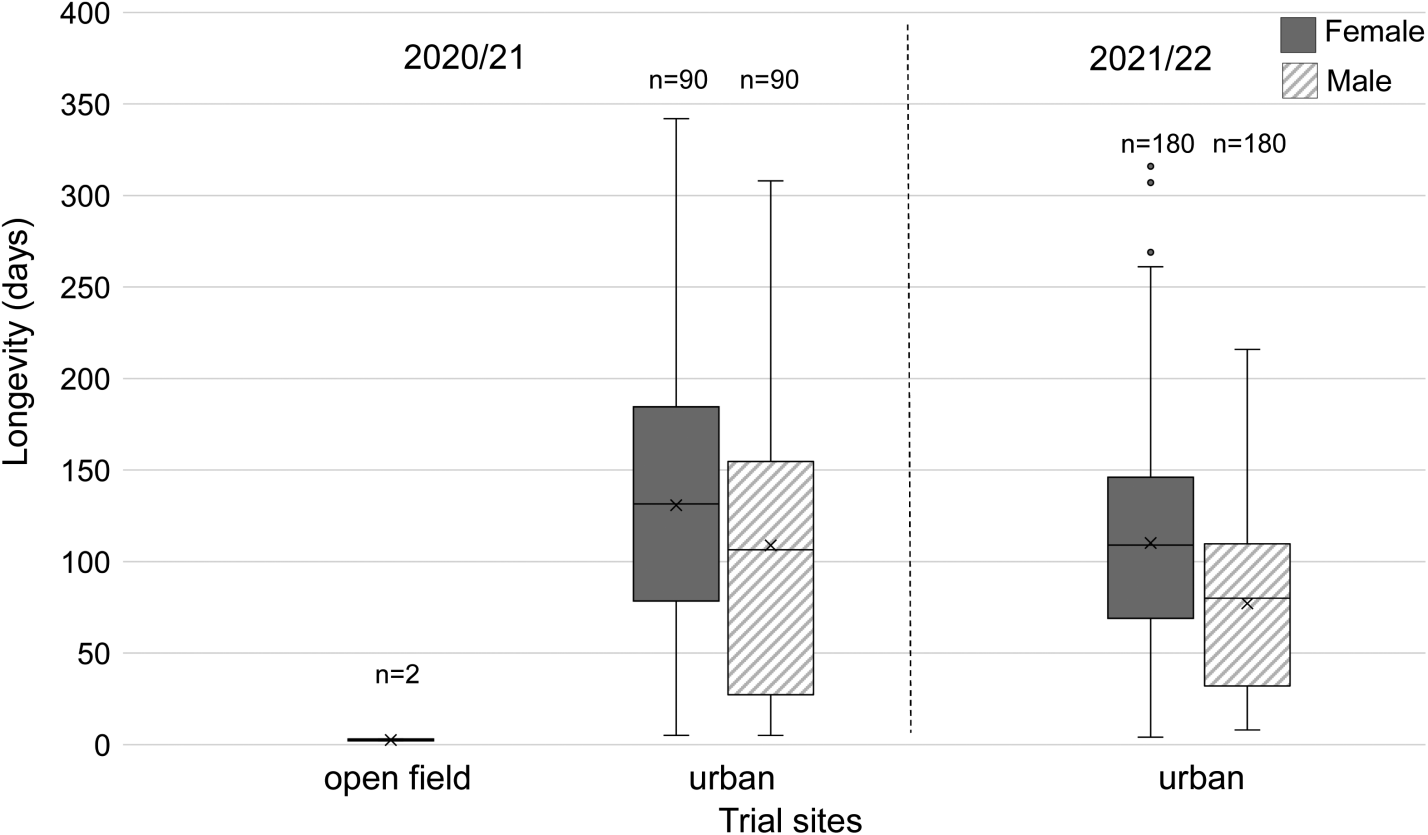
Longevity (days) of male and female *Ceratitis capitata* adults obtained from pupae exposed at different trial sites in Vienna, Austria during the seasons 2020/2021 and 2021/2022. No adults emerged in 2021/22 from overwintering pupae under open field conditions. Boxplots include the median (line), mean (x), and the first and third quartile; whiskers indicate the minimum/maximum value. A data point is considered an outlier (gray dots), if it exceeds a distance of ± the 1.5-fold interquartile range from the first/third quartile. In both seasons females lived longer than males (*P* < 0.05).

Longevity of adults that emerged from pupae under urban conditions differed between the 2 seasons (Fig. 4). The longest recorded lifespans (oldest adult) were 342 d (♀) (2020/2021) and 316 d (♀) (2021/2022). During both seasons, female longevity was longer than that of male (*χ*^2^ = 4.042, df = 1, *P *= 0.044; *χ*^2^ = 30.290, df = 1, *P *< 0.001 for the 2020/2021 and 2021/2022 season, respectively). Establishment date was also a significant predictor of adult longevity (*χ*^2^ = 6.994, df = 2, *P *= 0.030; *χ*^2^ = 10.081, df = 2, *P *= 0.006 for the 2020/2021 and 2021/2022 season, respectively). The earlier the establishment date the longer the longevity of adults. In total, 62.8% (2020/21) and 38.9% (2021/2022) of adults obtained from pupa survived at this site at least until the beginning of March of the following year. Twenty-seven point 2 (27.2%) (2020/2021) and 9.2% (2021/2022) lived until the beginning of May (start of subsequent fruiting season) or beyond.

**Fig. 5.**
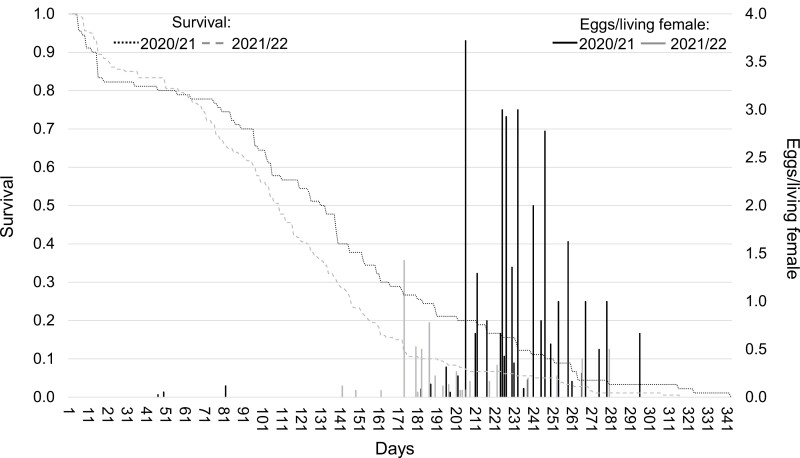
Cumulative survival rates (lines; absolute lifespan (days)) and fecundity (columns; eggs per living female) of *Ceratitis capitata* adults from overwintering pupae trials at the trial site urban in Vienna, Austria during the seasons 2020/2021 and 2021/2022.

The proportion of females engaged in oviposition events was lower (6.1%) in 2021/22 than in 2020/21 (17.8%) (*x*^2^ = 7.8, df = 1, *P* = 0.0055). The main oviposition period of the first season (96.5% of all eggs) lasted from June until September 2021, while few eggs were deposited in December 2020 and February 2021 (Fig. 5). In contrast, the main oviposition period of the second season started already in March 2022, with the majority of eggs being counted before June 2022 (73.3%).

The average fecundity (number of eggs per female) was 0.6 ± 0.3 (SE) and 4.4 ± 1.5 (SE) in 2021/2022 and 2020/2021, respectively. The establishment date of pupae was not a predictor of the female preoviposition period (2020/2021; *χ*^2^ = 5.003, df = 2, *P *= 0.082; 2021/2022; *χ*^2^ = 5.886, df = 2, *P* = 0.053), oviposition period (2020/2021; *χ*^2^ = 3.445, df = 2, *P* = 0.179; 2021/2022; *χ*^2^ = 1.336, df = 2, *P *= 0.513), and post-oviposition period (2020/2021; *χ*^2^ = 2.619, df = 2, *P* = 0.270; 2021/2022; *χ*^2^ = 3.195, df = 2, *P* = 0.202).

### Larvae Overwintering Trials

At the control under laboratory conditions, infested apples from the larvae overwintering trials yielded on average 18.2 ± 0.7 (SE) (2020/21) and 15.8 ± 1.2 (SE) (2021/22) pupae per apple. No larva completed development in open field conditions. In urban conditions, 4.6 ± 0.8 (SE) (2020/2021) and 6.7 ± 0.7 (SE) (2021/2022) pupae were obtained from the infested fruits.

Mean development rate from larvae to pupae was shorter at the earlier establishment dates than at the last (*χ*^2^ = 92.446, df = 2, *P *< 0.001; *χ*^2^ = 7.525, df = 2, *P = *0.023 for the 2020/2021 and 2021/2022 season, respectively).

Adult emergence rate at the control was nearly 90% in the first and over 70% in the second season. None of the pupae developed under urban conditions in 2020/2021 resulted in emerging adults, and only a few adults (2.0%) emerged in 2021/2022. All of those specimens hatched after a development time of 34–41 d, no adult emerged before or after this period. Their average lifespan was 16.7 ± 6.3 d (SE) (♀) and 23.6 ± 5.8 d (SE) (♂). None of the females laid eggs during the trial period and only 3 adults (2♂♂, 1♀) survived the winter months living until 23 March 2022.

## Discussion

Since 2010, annual occurrences of *C. capitata* have been recorded in Austria (Egartner et al. 2019), raising the question of the origin of those flies. Although recurrent entries of immature stages with infested fruits are presumably one of the main sources, the detections in consecutive years in the same area indicated a potential for overwintering of individuals in Austria. To our knowledge, this is the first time a study has focused on the overwintering capability of various medfly life stages this far north (48°N) beyond its natural habitat, which has previously been considered to be up to the 41° north latitude (Papadopoulos et al. 1996, 1998, Peñarrubia-María et al. 2012).

Our results revealed that none of the tested *C. capitata* life stage was able to survive the Austrian winter conditions in the open field. At the end of November, minimum temperatures started to drop below 0 °C in both trial seasons and the mortality rate of adults exposed to outdoor conditions increased accordingly. Eventually, all adults were dead by the beginning of January at the latest.

The adult stage is considered the most vulnerable life stage of *C. capitata* to low temperatures (Nyamukondiwa et al. 2013, Manrakhan et al. 2022). The critical thermal minima (CT_min_), defined as the temperature at which each individual insect loses coordinated muscle function, consequently also losing the ability to respond to mild stimuli (e.g., prodding), for *C. capitata* was measured between 5.4 and 6.6 °C with age and feeding status dependent variations (Nyamukondiwa and Terblanche 2009). While after a mild cold exposure, insects can regain muscle function after returning to warmer temperatures (chill-coma recovery), the mean maximum temperatures in the open field in Vienna during December and January were only 4.6–5.4 °C (2020/21) and 5.4–6.0 °C (2021/22). Consequently, with the beginning of winter, adults were regularly exposed to temperatures below the CT_min_ over an extended period presumably leading to chill-coma-induced immobility. Reduced in their movements, adults were likely no longer able to secure their demands for nutrient and hydration and died from dehydration or starvation. In nature, this immobility would also make the flies more vulnerable to predation. However, a direct death from freezing injuries is also likely. Various studies have investigated the lethal thermal limits of *C. capitata*. Nyamukondiwa et al. (2013) reported a mortality rate of 50% of adults after 8 h exposure to −0.61 °C, and 90% at −2.58 °C. Papadogiorgou et al. (2023) estimated the lower lethal temperature (LLT) at −5.5 °C for *C. capitata* after 1 h of exposure. During both overwintering seasons, temperatures frequently dropped below 0 °C (on ~30% of the days from November to January), exposing the adult flies extensively to their lethal thermal limits. Additionally, trials with different *C. capitata* populations showed that the lower thermal limit has a high plasticity, depending on origin of the flies and prior acclimation to lower temperatures (Papadogiorgou et al. 2023). For example, an acclimation at 10 °C for 5 d before the trials shifted the LLT to a lower level, resulting in a higher survival rate compared to an acclimation at temperatures >10 °C. In our case, adult flies were exposed to open field conditions in autumn, when the temperatures were still above 15 °C and flies had enough time for acclimation. However, considering the extended exposure time to low temperatures in the open field, a prior acclimation can only delay death, but not prevent it.

Our findings are congruent with overwintering studies from other regions with subfreezing temperatures in Thessaloniki, Greece (40°N) (Papadopoulos et al. 1996) and Girona, Spain (42°N) (Peñarrubia-María et al. 2012), where adults also failed to survive the winter period. Additionally, surveillance programs conducted in Greece showed that on the Chios islands (38°N) adults are trapped only until January (Katsoyannos et al. 1998) and in Thessaloniki until December (Papadopoulos et al. 2001). Successful overwintering of adults was reported just from areas with mild winters like Crete (35°N) (Mavrikakis et al. 2000) or West-Australia (32°S) (Rahman and Broughton 2019). Rahman and Broughton (2019) also showed that adding a live branch to the experimental cages can improve the survivability of flies by providing protection against adverse factors such as rain, wind, etc. In nature, flies seek refuge in warmer climate during winter, for example, under citrus canopy, where temperature is 1–2 °C higher than in open air (De Lima and Broughton 2001). In the current study, cages with adults were protected from rain and wind by placing them in a roofed building with double-netted walls.

Similar to the adult stage, larvae and pupae were not able to survive in the open field either. Not 1 pupa developed from the infested apples of the larvae trials and very few adults hatched from the exposed pupae. All of them emerged before the beginning of winter in October and November 2020, and died soon afterwards.

The impact of cold climate conditions on an organism is dependent upon various factors, including temperature, duration of exposure, or thermal developmental thresholds and their interactions. Several studies have investigated the developmental thresholds for eggs, larvae, and pupae with varying results. Conti (1988) reported 11 °C for pupa, Shoukry and Hafez (1979) 5 °C for larva and 13 °C for pupa, while De Lima (2008) estimated 9.3, 11.1, and 8.4 °C for egg, larva, and pupa stage and Duyck and Quilici (2002) 11.6, 10.2, and 11.2 °C, respectively. Lower lethal limits (LLT_90;_ 90% dead after 8 h exposure) of *C. capitata* were studied by Nyamukondiwa et al. (2013) with −3.01 °C for larva and −6.25 °C for pupa. While an exposure to lesser cold temperatures (−0.61 °C for 7 d) over a prolonged time could also result in a high larvae mortality rate (99%) (Manrakhan et al. 2022). However, even if immatures survive cold periods, their ability for further development are severely limited (Gazit et al. 2014, Hallman et al. 2019). The mean minimum temperatures in the open field from December to February ranged from −0.6 to 1.6 °C in 2020/21 and 0.0 to 2.3 °C in 2021/22. Additionally, during Field Control, infested apples were regularly transferred from the open field to laboratory conditions to check, if larvae within the apples were still alive and able to pupate. However, no larva was able to develop to pupal stage after 24 November 2021 under these conditions. This was also the day when subfreezing temperatures occurred for the first time at this test site and remained for several hours. Given that, the immatures in our studies were probably either inhibited from completing their development or died from the experience of continuous lethal temperatures. Similar observations were made by Rigamonti (2004), while investigating the overwintering potential of larvae in apples in Lombardy (45°N) with the conclusion that all larvae died after the first period of intense coldness in the open field.

The same results were observed for the pupal stage. Ali Ahmed et al. (2007) reported that adult *C. capitata* had the highest emergence rate with 69.3% when pupae were buried between 0 and 2 cm. While pupae buried from 2 to 10 cm still emerged at a rate of 54–58%. These trials were conducted in the laboratory at 30.5 ± 1 °C and a relative humidity of 50 ± 5%. We hypothesized that in the open field a greater depth would improve the survival chances of pupae during an Austrian winter. Indeed, temperatures at 10 cm depth, where tested pupae were buried, dropped below 0 °C on only 7 d in 2020/21, and none in 2021/22, respectively. In contrast, the minimum air temperature for the same period was on 54 (2020/21) and 56 (2021/22) days below zero. However, although the soil acted as a thermal buffer, protecting the pupae mostly from being exposed to subfreezing temperatures, temperatures were still insufficient for completing adult development. Several studies reported similar results of pupae failing to survive the winter period at the northern frontier of distribution (Papadopoulos et al. 1996, Rigamonti 2004).

However, in case of the larvae, studies in Thessaloniki, Greece (40°N) showed that despite the occasional prevalence of subfreezing temperatures a successful overwintering of larvae is possible as first and second instars in apples (Papadopoulos et al. 1996, 1998). A reason for the diverging results could lay in the genetic traits of the different medfly populations in Greece and Austria and the severity of winter. A comparison of *C. capitata* adults from Vienna and Thessaloniki by Moraiti et al. (2022) revealed that Viennese flies had a higher chill-coma recovery time and lower post-recovery survival rate compared to adults from Greece, resulting in a lesser chill-tolerant population. In addition, the winter season is shorter and warmer in Thessaloniki, Greece, which is a coast city in the Aegean Sea, where the frequency of subfreezing temperature events is lower compared to Vienna.

While *C. capitata* is established in the area of Thessaloniki, and apparently may have gone through an adaptation to the specific climate conditions that lay within northern limits of its geographic range to the Balkan Peninsula, the origin of the flies detected every year in Vienna is still unclear. Annual reintroductions of *C. capitata* propagules in Austria through trade of fresh fruit and human travel from warmer regions are likely. Adaptation to cold temperatures is therefore unlikely in this invasion scenario and therefore no cold tolerance of local flies may have developed. Genetic studies from Germany on the origin of trapped medflies from local surveillance programs revealed, that they were not part of an established population, but annually reintroduced from France and Croatia (König et al. 2022). Nonetheless, a careful inspection of the data given in König et al. (2022) reveals genetic relatedness of the German specimens and close association with neighboring French ones. Hence, temporal breeding populations and temporal overwintering cannot be excluded as well.

In recent years, there is a growing interest in the effects of urban heat islands on arthropods. Numerous studies have investigated the impact of those thermal hot pockets, where temperatures can be several degrees Celsius higher compared with nearby nonurban areas (Diamond and Martin 2020), on behavior, survival, and development of various species (Villalobos-Jiménez and Hassall 2017, Nguyen et al. 2018, Chick et al. 2019, Diamond and Martin 2021). Given the unique thermal conditions of those isolated areas, it is hypothesized that these heat islands may enable the survival of species that would otherwise not be able to endure the low temperatures during the winter period at higher latitudes. Buildings and structures in urban areas can also provide protected environments with more favorable climate conditions to overwintering organisms. Both of our study sites were placed within a suburban surrounding in the periphery of Vienna, where they could benefit from higher temperatures compared with the nonurban areas outside of the city. At the test site urban conditions, we additionally investigated the influence of a protected habitat on the survival chances during the winter period. Comparable conditions can be found in Vienna or in houses at the countryside, for example, in the cellars of garden houses in urban allotments, where fruits from gardens are stored. Similar conditions can occur across many countries of the temperate zone, where they potentially could act as shelter during periods of unfavorable climate conditions.

And indeed, our results showed that medfly, when exposed to such an environment, is able to endure the Austrian winter period. Larvae from infested apples were able to pupate, emerge as adults, and at least in 1 season survive until March of the following year. Several flies of the adult trials lived until May or beyond, with some adults showing a prolonged lifespan extending to autumn of the year after trial start.

Males of our control group outlived females, which was comparable to previous studies conducted under similar laboratory conditions with populations from different parts of the world, even if female individuals in other studies often were found to be the last to die within a cohort (Carey et al. 1995, Diamantidis et al. 2009). However, while globally male medflies seem to have a longer life expectancy, females from our adult and pupa trials under urban conditions outlived males in both overwintering seasons. Similar observations were made by Rahman and Broughton (2019). Therefore, while urban conditions were advantageous for both sexes in terms of an increased average survival time, these seem to have resulted in an even greater advantage for the females.

The influence of external conditions, such as the available diet or the absence of sexual partners under artificial conditions, have previously shown serious effects on the lifespan of females. For example, the composition of the diet, especially the absence of the required proteins, can have a major impact on reproduction and longevity (Carey et al. 1998, 2002). The possibility of medfly females to bridge unfavorable periods by reducing their rate of aging allows the pest to better persist under unfavorable conditions (Carey 1999). It is conceivable that the females under urban conditions, which produced comparably few or even no eggs, had to use fewer resources for reproduction than it would have been the case under laboratory conditions or in the wild, which could have increased their life expectancy. The given conditions could also have caused females to enter the “waiting mode” (Carey 1999) in which reproduction is low but survival high. Eventually, the increase of the temperatures in spring may have led the females to switch to the reproductive mode, which again could have an effect on the longevity, as this is more beneficial than remaining in 1 mode exclusively (Carey et al. 1998). Consequently, the resulting positive influence of the given conditions may have had a greater impact on female longevity than on the males.

Of great importance, of course, is that the trials on the lifespan and reproduction potential of adults obtained from the pupae trials revealed their principal capability to produce (albeit few) eggs after the winter period and therefore to form a new generation in the subsequent fruiting season. Our results are consistent with studies from Lombardy, Italia (45°N) (Rigamonti 2004) examining the overwintering potential of adult medflies in a preserved indoor environment. However, the trials presented in this study only examined the oviposition capability of adults that emerged from larvae and/or pupae trials, but not from overwintering adults. Future experiments should include this parameter for the adult stage (Rahman and Broughton 2019) by offering regularly fruits for oviposition and checking for eggs/larvae.

Indeed, a successful indoor overwintering would require various factors such as adult flies actively seeking shelter or the storage of infested fruits at these sites. Additionally, even if sufficient food and water is provided, probably only a reduced number of flies would be able to survive until spring. However, given the generally high fecundity of the flies (White and Elson-Harris 1992), even if this was clearly reduced under our conditions with only few eggs oviposited in spring, it can be assumed that indoor overwintering could represent a way for winter survival of a (small) population in regions where outdoor conditions would not allow this.

In the current study, the overwintering potential of *C. capitata* was tested for the larval, pupal, and adult life stage, but not for the egg stage. Trials by Papadopoulos et al. (1996, 1998) in Northern Greece (40°N) concluded that the egg stage is not able to survive a cold winter, only the early larval instars. In contrast, Rahman and Broughton (2019) reported that in Western Australia (32°S) eggs (1–6%) were able to survive winter and emerge as adults. However, winter in this region was very mild with temperature only briefly dropping below 0 °C on some occasions and absolute maximum temperatures reaching 19–26 °C in the same period.

Another important factor for a potential establishment of *C. capitata* in Austria is climate change. Since 1880 the average air temperature in Austria has increased by roughly 2 °C, which is twice as much as the global average (Kromp-Kolb et al. 2014). The same authors reported that an additional increase of 1–2 °C is projected until 2050. At the same time, winter days with temperatures below −5 °C will constantly decline (GeoSphere 2024). The mean minimum winter temperature (December–February) in Vienna from 1990 to 2020 was −1.33 to −0.25 °C (Historical weather data from Hohe Warte; 48°15ʹ7″N, 16°21ʹ24″O; 214 m above sea level) (Stadt Wien 2024). However, during our trial period, we measured −0.6 to 1.6 °C in 2020/21 and 0.0 to 2.3 °C in 2021/22 in the same period, meaning that the winter periods at our trials were already warmer than the average last 3 decades. And, presumably, temperatures will increase even further in the near future. With this in mind, our results only provide a snapshot of the current situation, as climatic conditions for an establishment of *C. capitata* in Austria will change over the next decades. In conclusion, our results demonstrate that *C. capitata* is currently not able to survive the winter period in Austria under natural conditions in the open field. However, we present a scenario in which the Mediterranean fruit fly could survive adverse outdoor conditions in regions beyond its northern range limits.

## Data Availability

All data will become available upon request and will be uploaded in an open access folder of the FF-IPM project.

## References

[CIT0002] Ali Ahmed A , Soltani N, Kellouche A, et al. 2007. Effects of the soil texture and the burying depth of the larvae on some biological parameters of *Ceratitis capitata* (Diptera: Trypetidae). Afr. J. Agric. Res. 2(3):105–111.

[CIT0003] APHIS—Animal and Plant Health Inspection Service USDA. 2024. U.S. regulated plant pest list. [accessed 2024 Mar 7] https://www.aphis.usda.gov/plant-imports/regulated-pest-list.

[CIT0004] Badii K , Billah M, Afreh-Nuamah K, et al. 2015. Review of the pest status, economic impact and management of fruit-infesting flies (Diptera: Tephritidae) in Africa. Afr. J. Agric. Res 10(12):1488. 10.5897/AJAR2014.9278

[CIT0005] Beck HE , Zimmermann NE, McVicar TR, et al. 2018. Present and future Köppen-Geiger climate classification maps at 1-km resolution. Sci. Data 5(1):1–12. 10.1038/sdata.2018.214 30375988 PMC6207062

[CIT0006] Böhm H. 1958. Zum Vorkommen der Mittelmeerfruchtfliege Ceratitis capitata Wied., im Wiener Obstbaugebiet. Pflanzenschutzberichte XXI(9/10):129–158. Band.

[CIT0007] Boller E. 1958. *Rhagoletis cerasi* and *Ceratitis capitata*. In: Singh P and Moore RF, editors. Handbook of insect rearing, Vol. 2. Amsterdam: Elsevier; ; p. 135–144.

[CIT0008] Bonizzoni M , Guglielmino C, Smallridge C, et al. 2004. On the origins of medfly invasion and expansion in Australia. Mol. Ecol. 13(16):3845–3855. 10.1111/j.1365-294X.2004.02371.x 15548296

[CIT0009] Carey JR. 1999. How Mediterranean fruit flies resist aging, live long and remain fertile. In: Robine JM, Forette B, Franceschi C, Allard M, editors. The paradoxes of longevity. Berlin, Heidelberg: Springer; p. 23–24.

[CIT0010] Carey JR , Liedo P, Orozco D, et al. 1995. A male-female longevity paradox in medfly cohorts. J. Anim. Ecol. 64(1):107–116. 10.2307/5831

[CIT0011] Carey JR , Liedo P, Müller HG, et al. 1998. Dual modes of aging in Mediterranean fruit fly females. Science 281(5379):996–998. 10.1126/science.281.5379.996 9703516

[CIT0012] Carey JR , Liedo P, Harshman L, et al. 2002. A mortality cost of virginity at older ages in female Mediterranean Fruit Flies. Exp. Gerontol. 37(4):507–512. 10.1016/s0531-5565(01)00230-3 11830353

[CIT0013] Chick LD , Strickler SA, Perez A, et al. 2019. Urban heat islands advance the timing of reproduction in a social insect. J. Therm. Biol. 80:119–125. 10.1016/j.jtherbio.2019.01.004 30784475

[CIT0015] Conti B. 1988. Effects of abiotic factors on *Ceratitis capitata* (Wied.) (Diptera Tephritidae). III: larval and total development under constant temperatures. Frustula Entomol. 11:157–169.

[CIT0016] Cullum JP , Nixon LJ, Morrison WR, et al. 2020. Influence of landscape factors and abiotic conditions on dispersal behavior and overwintering site selection by *Halyomorpha halys* (Hemiptera: Pentatomidae). J. Econ. Entomol. 113(4):2016–2021. 10.1093/jee/toaa077 32435807

[CIT0017] De Lima CPF. 2008. Area wide management of Mediterranean fruit fly in Australia. Acta Hortic. 803(803):51–60. 10.17660/actahortic.2008.803.5

[CIT0018] De Lima CPF , Broughton S. 2001. Investigation of fruit damage in citrus in South West Western Australia. Sydney, Australia: Horticultural Research and Development Corporation; p. 23.

[CIT0019] De Meyer M , Copeland RS, Lux SA, et al. 2002. Annotated check list of host plants for Afrotropical fruit flies (Diptera: Tephritidae) of the genus Ceratitis, Vol. 27, Tervuren, Belgium: Royal Museum for Central Africa / Musée Royal de l’Afrique Centrale; p. 91.

[CIT0020] Deschepper P , Todd TN, Virgilio M, et al. 2021. Looking at the big picture: worldwide population structure and range expansion of the cosmopolitan pest *Ceratitis capitata* (Diptera, Tephritidae). Biol. Invasions 23(11):3529–3543. 10.1007/s10530-021-02595-4

[CIT0021] Diamantidis AD , Papadopoulos NT, Nakas CT, et al. 2009. Life history evolution in a globally invading tephritid: patterns of survival and reproduction in medflies from six world regions. Biol. J. Linn. Soc. 97(1):106–117. 10.1111/j.1095-8312.2009.01178.x

[CIT0022] Diamond SE , Martin RA. 2020. Evolutionary consequences of the urban heat island. In: Szulkin M, Munshi-South J, Charmantier A, editors. Urban evolutionary biology. New York, USA: Oxford University Press; p. 91–110.

[CIT0023] Diamond SE , Martin RA. 2021. Physiological adaptation to cities as a proxy to forecast global-scale responses to climate change. J. Exp. Biol. 224(pt suppl 1):jeb229336. 10.1242/jeb.229336 33627462

[CIT0024] Duyck PF , Quilici S. 2002. Survival and development of different life stages of three *Ceratitis* spp. (Diptera: Tephritidae) reared at five constant temperatures. Bull. Entomol. Res. 92(6):461–469. 10.1079/ber2002188 17598297

[CIT0025] Duyck PF , David P, Pavoine S, et al. 2008. Can host-range allow niche differentiation of invasive polyphagous fruit flies (Diptera: Tephritidae) in La Réunion? Ecol. Entomol. 33(4):439–452. 10.1111/j.1365-2311.2008.00989.x

[CIT0026] Egartner A , Lethmayer C, Gottsberger RA, et al. 2019. Recent records of the Mediterranean fruit fly, *Ceratitis capitata* (Tephritidae, Diptera) in Austria. IOBC-WPRS Bull. 146:143–152.

[CIT0027] EPPO. 2024. EPPO global database. [accessed 2024 Jun 7] https://gd.eppo.int.

[CIT0028] EUROPHYT—European Union Notification System for Plant Health Interceptions. 2015. Interceptions of commodities imported into the EU or Switzerland with harmful organism(s)—Monthly Report May 2015. [accessed 2024 Jun 4] https://food.ec.europa.eu/document/download/78070e91-b115-43c6-8c6e-59b3906b6b87_en?filename=ph_biosec_europhyt-interceptions-2015-05.pdf.

[CIT0029] EUROPHYT—European Union Notification System for Plant Health Interceptions. 2017. Interceptions of commodities imported into the EU or Switzerland with harmful organism(s)—Monthly Report August 2017. [accessed 2024 Jun 4] https://food.ec.europa.eu/document/download/3ed1bc11-014f-42cd-a70a-79cd4e04a6a1_en?filename=ph_biosec_europhyt-interceptions-2017-08.pdf.

[CIT0030] Fischer-Colbrie P , Bush-Petersen E. 1989. Temperate Europe and West Asia. In: Robinson AS, Hooper G, editors. Fruit flies: their biology, natural enemies and control, Vol. 3A. Amsterdam: Elsevier; p. 91–99.

[CIT0031] Gazit Y , Akiva R, Gavriel S. 2014. Cold tolerance of the Mediterranean fruit fly in date and mandarin. J. Econ. Entomol. 107(5):1745–1750. 10.1603/EC14050 26309262

[CIT0032] GeoSphere Austria—Bundesanstalt für Geologie, Geophysik, Klimatologie und Meteorologie. 2024. [accessed 2024 Jun 9] https://www.zamg.ac.at/cms/de/klima/informationsportal-klimawandel/klimavergangenheit/neoklima/hitze.

[CIT0033] Gilioli G , Sperandio G, Colturato M, et al. 2022. Non-linear physiological responses to climate change: the case of *Ceratitis capitata* distribution and abundance in Europe. Biol. Invasions 24(1):261–279. 10.1007/s10530-021-02639-9

[CIT0034] Glaeser G. 1979. Bericht über das Auftreten wichtiger Krankheiten und Schädlinge an Kulturpflanzen in Österreich im Jahre 1977. Pflanzenschutzberichte 45(7/12):153–164.

[CIT0035] Grout TG , Stoltz KC. 2007. Developmental rates at constant temperatures of three economically important *Ceratitis* spp. (Diptera: Tephritidae) from Southern Africa. Environ. Entomol. 36(6):1310–1317. 10.1603/0046-225x(2007)36[1310:dracto]2.0.co;2 18284758

[CIT0036] Hallman GJ , Wang L, Uzel GD, et al. 2019. Comparison of populations of *Ceratitis capitata* (Diptera: Tephritidae) from three continents for susceptibility to cold Phytosanitary treatment and implications for generic cold treatments. J. Econ. Entomol. 112(1):127–133. 10.1093/jee/toy331 30346545

[CIT0037] IBM Corp. Released 2019. IBM SPSS Statistics for Windows, Version 26.0. Armonk, NY: IBM Corp.

[CIT0038] IPPC Secretariat. 2021. Catalogue of quarantine pests for import plants to China (update20210409). [accessed 2024 Jun 10] https://assets.ippc.int/static/media/files/reportingobligation/2022/02/21/Catalogue_of_Quarantine_Pests_for_Import_Plants_to_China_update20210409.pdf.

[CIT0039] Israely N , Ritte U, Oman SD. 2004. Inability of *Ceratitis capitata* (Diptera: Tephritidae) to overwinter in the Judean Hills. J. Econ. Entomol. 97(1):33–42. 10.1093/jee/97.1.33 14998125

[CIT0040] Katsoyannos BI , Kouloussis NA, Carey JR. 1998. Seasonal and annual occurrence of Mediterranean fruit flies (Diptera: Tephritidae) on Chios Island, Greece: differences between two neighboring citrus orchards. Ann. Entomol. Soc. Am. 91(1):43–51. 10.1093/aesa/91.1.43

[CIT0041] König S , Steinmöller S, Baufeld P. 2022. Origin and potential for overwintering of *Ceratitis capitata* (Wiedemann) captured in an official survey in Germany. J. Plant Dis. Prot. 129(5):1201–1215. 10.1007/s41348-022-00605-8

[CIT0042] Kromp-Kolb H , Nakicenovic N, Pawloff A, et al. 2014. Austrian assessment report climate change 2014 (AAR14): synopsis. Main findings. Vienna, Austria: Austrian Academy of Sciences Press.

[CIT0043] Liquido N , McQuate G, Hanlin M, et al. 2020. Host plants of the Mediterranean fruit fly, *Ceratitis capitata* (Wiedemann) Version 4.0. USDA Compendium of Fruit Fly Host Information (CoFFHI). [accessed 2023 Apr 9] https://coffhi.cphst.org/.

[CIT0044] MAFF—Ministry of Agriculture, Forestry and Fisheries (Japan). 2022. Quarantine Pest List Annexed Table 1 of the Ordinance for Enforcement of the Plant Protection Act. [accessed 2024 Jun 6] https://www.maff.go.jp/pps/j/law/houki/shorei/E_Annexed_Table1.html.

[CIT0045] Manrakhan A , Daneel JH, Stephen PR, et al. 2022. Cold tolerance of immature stages of *Ceratitis capitata* and *Bactrocera dorsalis* (Diptera: Tephritidae). J. Econ. Entomol. 115(2):482–492. 10.1093/jee/toab263 35024832

[CIT0046] Mavrikakis PG , Economopoulos AP, Carey JR. 2000. Continuous winter reproduction and growth of the Mediterranean fruit fly (Diptera: Tephritidae) in Heraklion, Crete, southern Greece. Environ. Entomol. 29(6):1180–1187. 10.1603/0046-225x-29.6.1180

[CIT0047] Meixner M , McPheron B, Silva J, et al. 2002. The Mediterranean fruit fly in California: evidence for multiple introductions and persistent populations based on microsatellite and mitochondrial DNA variability. Mol. Ecol. 11(5):891–899. 10.1046/j.1365-294X.2002.01488.x 11975705

[CIT0048] Moraiti CA , Verykouki E, Papadopoulos NT. 2022. Chill coma recovery of *Ceratitis capitata* adults across the Northern Hemisphere. Sci. Rep. 12(1):17555. 10.1038/s41598-022-21340-y 36266456 PMC9585097

[CIT0049] Nguyen HQ , Andersen DK, Kim Y, et al. 2018. Urban heat island effect on cicada densities in metropolitan Seoul. PeerJ 6:e4238. 10.7717/peerj.4238 29340243 PMC5768176

[CIT0050] Nyamukondiwa C , Terblanche JS. 2009. Thermal tolerance in adult Mediterranean and Natal fruit flies (*Ceratitis capitata* and *Ceratitis rosa*): effects of age, gender and feeding status. J. Therm. Biol. 34(8):406–414. 10.1016/j.jtherbio.2009.09.002

[CIT0051] Nyamukondiwa C , Kleynhans E, Terblanche JS. 2010. Phenotypic plasticity of thermal tolerance contributes to the invasion potential of Mediterranean fruit flies (*Ceratitis capitata*). Ecol. Entomol. 35(5):565–575. 10.1111/j.1365-2311.2010.01215.x

[CIT0052] Nyamukondiwa C , Weldon CW, Chown SL, et al. 2013. Thermal biology, population fluctuations and implications of temperature extremes for the management of two globally significant insect pests. J. Insect Physiol. 59(12):1199–1211. 10.1016/j.jinsphys.2013.09.004 24080125

[CIT0053] Papadogiorgou GD , Moraiti CA, Nestel D, et al. 2023. Acute cold stress and supercooling capacity of Mediterranean fruit fly populations across the Northern Hemisphere (Middle East and Europe). J. Insect Physiol. 147:104519. 10.1016/j.jinsphys.2023.104519 37121467

[CIT0054] Papadopoulos NT. 2014. Fruit fly invasion: historical, biological, economic aspects and management. In: Shelly T, Epsky N, Jang E, Reyes-Flores J, Vargas R, editors. Trapping and the detection, control, and regulation of Tephritid fruit flies. Dordrecht: Springer; p. 219–252. 10.1007/978-94-017-9193-9_7

[CIT0055] Papadopoulos NT , Carey JR, Katsoyannos BI, et al. 1996. Overwintering of the Mediterranean fruit fly (Diptera: Tephritidae) in northern Greece. Ann. Entomol. Soc. Am. 89(4):526–534. 10.1093/aesa/89.4.526

[CIT0056] Papadopoulos NT , Katsoyannos BI, Carey JR. 1998. Temporal changes in the composition of the overwintering larval population of the Mediterranean Fruit Fly (Diptera: Tephritidae) in Northern Greece. Ann. Entomol. Soc. Am. 91(4):430–434. 10.1093/aesa/91.4.430

[CIT0057] Papadopoulos NT , Katsoyannos BI, Carey JR, et al. 2001. Seasonal and annual occurrence of the Mediterranean fruit fly (Diptera: Tephritidae) in Northern Greece. Ann. Entomol. Soc. Am. 94(1):41–50. 10.1603/0013-8746(2001)094[0041:saaoot]2.0.co;2

[CIT0058] Papadopoulos NT , Plant RE, Carey JR. 2013. From trickle to flood: the large-scale, cryptic invasion of California by tropical fruit flies. Proc. Biol. Sci. 280(1768):20131466. 10.1098/rspb.2013.1466 23926154 PMC3757976

[CIT0059] Peñarrubia-María E , Avilla J, Escudero-Colomar LA. 2012. Survival of wild adults of *Ceratitis capitata* (Wiedemann) under natural winter conditions in North East Spain. Psyche, 1:1–6. 10.1155/2012/497087 Article ID 497087.

[CIT0060] Rahman T , Broughton S. 2019. The survival of Mediterranean fruit fly (Diptera: Tephritidae) over winter in Western Australia. Environ. Entomol. 48(4):977–987. 10.1093/ee/nvz060 31120521

[CIT0061] Rigamonti IE. 2004. Contributions to the knowledge of *Ceratitis capitata* Wied. (Diptera, Tephritidae) in Northern Italy: II. Overwintering in Lombardy. Boll. Zool. Agrar. Bachic. 36(1):101–116.

[CIT0062] Shoukry A , Hafez M. 1979. Studies on the biology of the Mediterranean fruit fly *Ceratitis capitata*. Entomol. Exp. Appl. 26(1):33–39. 10.1111/j.1570-7458.1979.tb02894.x

[CIT0063] Siebert J , Cooper T. 1995. If medfly infestation triggered a trade ban: embargo on California produce would cause revenue, job loss. Calif. Agric. 49(4):7–12. 10.3733/ca.v049n04p7

[CIT0064] Stadt Wien—Magistrat Wien. 2024. Magistratsabteilung 23—Wirtschaft, Arbeit und Statistik. Monatliche Wetterdaten der Messstation Hohe Warte zu Lufttemperatur, Luftdruck, Bewoelkung, Windgeschwindigkeit und Niederschlag seit April 1872—Wien. [accessed 2024 Jun 6] https://www.data.gv.at/katalog/de/dataset/wetter-seit-1872-hohe-warte-wien#resources.

[CIT0065] State of California—CDFA. 2023. Preventative Release Program (Medfly). [accessed 2023 Jul 20] https://www.cdfa.ca.gov/plant/PDEP/prpinfo/.

[CIT0066] Szyniszewska AM , Tatem AJ. 2014. Global assessment of seasonal potential distribution of Mediterranean fruit fly, *Ceratitis capitata* (Diptera: Tephritidae). PLoS One 9(11):e111582. 10.1371/journal.pone.0111582 25375649 PMC4222914

[CIT0067] Szyniszewska AM , Bieszczak H, Kozyra K, et al. 2024. Evidence that recent climatic changes have expanded the potential geographical range of the Mediterranean fruit fly. Sci. Rep. 14(1):2515. 10.1038/s41598-024-52861-3 38291088 PMC10828498

[CIT0068] Villalobos-Jiménez G , Hassall C. 2017. Effects of the urban heat island on the phenology of Odonata in London, UK. Int. J. Biometeorol. 61(7):1337–1346. 10.1007/s00484-017-1311-7 28190181 PMC5486733

[CIT0069] Watzl O. 1931. Die Mittelmeerfruchtfliege oder Pfirsichfliege (*Ceratitis capitata* Wied.). Neuheiten auf dem Gebiete des Pflanzenschutzes Mittl 6:161–164.

[CIT0070] Watzl O. 1932. Über ein Auftreten der Mittelmeerfruchtfliege (*Ceratitis capitata* Wied.) in Wien. Die Gartenbauwissenschaft 6:445–455.

[CIT0071] Weldon CW , Boardman L, Marlin D, et al. 2016. Physiological mechanisms of dehydration tolerance contribute to the invasion potential of *Ceratitis capitata* (Wiedemann) (Diptera: Tephritidae) relative to its less widely distributed congeners. Front. Zool. 13(1):1–15. 10.1186/s12983-016-0147-z 27034703 PMC4815119

[CIT0072] White IM , Elson-Harris MM. 1992. Fruit flies of economic significance: their identification and bionomics. Wallingford, UK: CAB International; p. xii + 601.

